# Drug connectivity mapping and functional analysis reveal therapeutic small molecules that differentially modulate myelination

**DOI:** 10.1016/j.biopha.2021.112436

**Published:** 2022-01

**Authors:** A.D. Rivera, F. Pieropan, G. Williams, F. Calzolari, A.M. Butt, K. Azim

**Affiliations:** a Institute of Biomedical and Biomolecular Sciences, School of Pharmacy and Biomedical Sciences, University of Portsmouth, St Michael’s Building, White Swan Road, PO1 2DT Portsmouth, UK; b Section of Human Anatomy, Department of Neuroscience, University of Padua, Padua, Italy; c Wolfson Centre for Age-Related Diseases, King's College London, Guy's Campus, London, UK; d Research Group Adult Neurogenesis & Cellular Reprogramming Institute of Physiological Chemistry, University Medical Center, Johannes Gutenberg University Mainz, Hanns-Dieter-Hüsch-Weg 19, 55128 Mainz, Germany; e Department of Neurology, Neuroregeneration, Medical Faculty, Heinrich-Heine-University, Düsseldorf, Germany

**Keywords:** LINCS, The Library of Integrated Network-based Cellular Signatures, iNSCs, iPSC-derived NSCs, SVZ, subventricular zone, NSC, Neural stem cell, OPC, Oligodendrocyte progenitor cell, MOL, Myelinating oligodendrocyte, NFOL, Newly formed oligodendrocyte, TCN, Triciribine, L-LY29, Low concentration of LY294002, H-LY29, High concentration of LY294002, TAPs, Transiently amplifying progenitors, MS, Multiple Sclerosis, CNS, Central Nervous System, iPCS, induced Pluripotent Stem Cell, OL, Oligodendrocyte, Oligodendrogenesis, Oligodendrocyte, Pharmacogenomics, The Library of Integrated Network-Based Cellular Signatures/LINCS, Subventricular zone, Optic nerve, PI3K/Akt

## Abstract

Disruption or loss of oligodendrocytes (OLs) and myelin has devastating effects on CNS function and integrity, which occur in diverse neurological disorders, including Multiple Sclerosis (MS), Alzheimer’s disease and neuropsychiatric disorders. Hence, there is a need to develop new therapies that promote oligodendrocyte regeneration and myelin repair. A promising approach is drug repurposing, but most agents have potentially contrasting biological actions depending on the cellular context and their dose-dependent effects on intracellular pathways. Here, we have used a combined systems biology and neurobiological approach to identify compounds that exert positive and negative effects on oligodendroglia, depending on concentration. Notably, next generation pharmacogenomic analysis identified the PI3K/Akt modulator LY294002 as the most highly ranked small molecule with both pro- and anti-oligodendroglial concentration-dependent effects. We validated these *in silico* findings using multidisciplinary approaches to reveal a profoundly bipartite effect of LY294002 on the generation of OPCs and their differentiation into myelinating oligodendrocytes in both postnatal and adult contexts. Finally, we employed transcriptional profiling and signalling pathway activity assays to determine cell-specific mechanisms of action of LY294002 on oligodendrocytes and resolve optimal *in vivo* conditions required to promote myelin repair. These results demonstrate the power of multidisciplinary strategies in determining the therapeutic potential of small molecules in neurodegenerative disorders.

## Graphical Abstract

**Figure:**
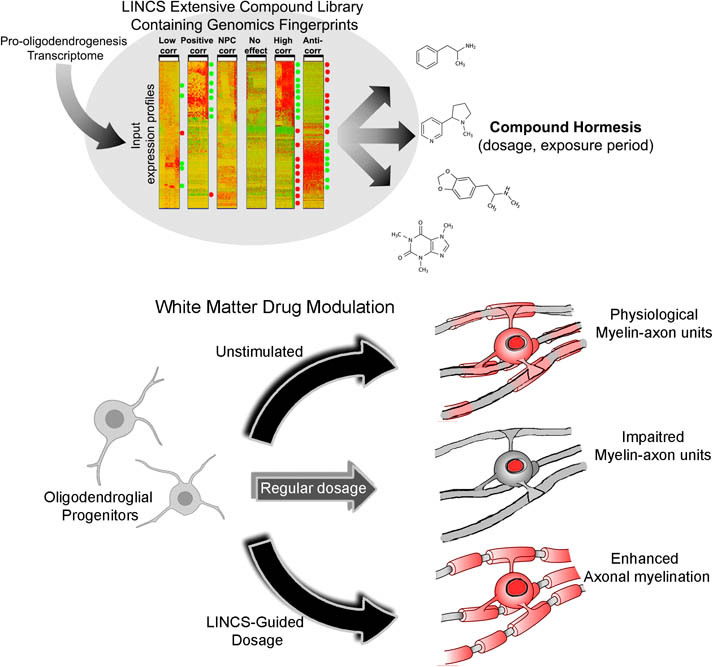

## 1 Introduction

In the Central Nervous System (CNS), myelin is produced by oligodendrocytes that are generated from oligodendrocyte progenitor cells (OPCs) throughout life [1]. During postnatal development, in addition to generating neural progenitors, neural stem cells (NSCs) of the subventricular zone (SVZ) also pass through a number of distinct differentiation stages to generate OPCs, which migrate throughout the forebrain and differentiate into myelinating oligodendrocytes (MOLs), in response to intrinsic and extracellular cues [2], [3]. In the adult brain, a significant population of endogenous OPCs persist throughout life and have the function of life-long generation of oligodendrocytes, which is essential for *de novo* myelination under physiological conditions and for myelin repair in pathology [4], [5]. In addition, the adult SVZ remains an important source of new OPCs to replenish endogenous populations, in particular following pathological demyelination [2], [6]. However, long-term repair ultimately fails due to a number of contributing factors including the decline in oligodendrocyte regeneration efficiency from both the SVZ [2], [7] and endogenous OPCs [8], [9], which severely impairs repair in numerous neuropathologies, including Multiple Sclerosis (MS) and age-related pathologies such as Alzheimer’s disease [9], [10]. Hence, there is a need for new therapies that promote OPC regeneration and repair.

Connectivity mapping has been used in multiple clinical areas to connect biology and drug discovery by exploiting transcriptional similarities across treatment conditions and cell states [11]. This strategy is a promising and direct approach to regulate neural cells and identify small molecules and transcriptional networks that have the potential to promote regeneration and repair in the CNS [2], [12], [13], [14]. However, agents identified by these new therapeutic approaches have potentially divergent biological actions depending on their dose- and time-dependent effects on intracellular regulatory pathways. Thus, determining the cell type-specific effects and precise mechanisms of action of small molecules on neural cells is essential for developing future therapeutic strategies to promote CNS repair. In the present study, we have identified small molecules with the potential to regulate oligodendrocyte (OL) generation by utilising a new comprehensive reference catalogue, LINCS (Library of Integrated Network-based Cellular Signatures), which hosts the gene expression phenotypes triggered by small molecules assayed at different concentrations across diverse cellular systems (https://clue.io). We identified a wide range of small molecules that targeted multiple regulatory pathways with the potential to both positively and negatively regulate OL differentiation. Significantly, the highest-ranking small molecule, LY294002, was predicted to have both pro- and anti-oligodendrogenic actions. At first glance, this appeared counter-intuitive, since LY294002 is a potent modulator of the PI3K/Akt signalling pathway, which is considered essential for OL differentiation and myelination [15], [16]. We validated the pharmacogenomic findings in multiple *in vivo* and *ex vivo* neurobiological models and demonstrated for the first time that LY294002 has a striking dose-dependent effect on oligodendrocytes, by severely disrupting biological pathways essential for oligodendrogenesis and myelinogenesis at high doses, but greatly stimulating OL generation and myelination at low doses. These developmental effects were recapitulated in isolated intact adult mouse optic nerves, thus providing relevance to adult white matter. Furthermore, using whole genome transcriptomics and biochemical assays, we clarified the cell type-specific mechanisms of action of low and high LY294002 concentrations on the oligodendroglial lineage. This study identifies strikingly contrasting dose-dependent actions of small molecules on neural cells, which would not otherwise be predicted from their known effects, highlighting the potential of systems biology approaches to guide the development of novel therapeutic strategies using small molecules to promote CNS repair.

## 2 Methods

### 2.1 Curation of oligodendroglial hallmark genes from previous studies

Our previous *in silico* screening for therapeutic agents capable of altering developmental myelination was performed using first generation of drug connectivity mapping [2], [17]. The LINCS [11] (https://clue.io), consists of a larger and cell type-specific resource, comprising 1.3 million expression profiles obtained from over 90 cell lines/induced Pluripotent Stem Cell (iPSC)-derived cells, of which the iPSC-derived Neural Stem Cells (iNSCs) drug-induced expression profiles were interrogated. Oligodendrogenesis-associated signatures were compiled using previously generated bulk and single-cell transcriptomic postnatal and adult OL lineage datasets [18], [19], [20], together with previously curated ‘pro-oligodendrogenesis’ signatures [2]. Genes deemed significantly and differentially expressed during OL differentiation in these datasets (< 5% false discovery rate; > 1.8 fold change) were standardised into Boolean values, while genes commonly expressed within these datasets were removed. The resulting list of 1170 genes comprised the essential landmark genes which define the later stages in the OL lineage as positive values, whereas those in the negative ranges are expressed in dorsal NSCs/transiently amplifying progenitors (TAPs) and in the earliest stages of OLs [2], [13], [19], [21].

### 2.2 Interrogating the LINCS-database for small molecule acquisition and defining their mechanisms of action on OL lineage cells

The LINCs resource L1000FWD (https://amp.pharm.mssm.edu/l1000fwd/#) was used to process expression signatures to query the IPSC-NPC datasets. The R package g:profiler via RStudio was used to convert mouse gene symbols to human. The following link contains the final expression profiles and derived small molecules: https://amp.pharm.mssm.edu/l1000fwd/result/5e638d9763095f00340d5b7e. A number of small molecules (each tested in triplicate) within the database have been tested under more than one dose/duration condition, thus enabling the dissection of potential concentration-dependent and temporal effects. Duplicates within the top 25 for the pro-oligodendroglial or top 15 anti-correlating (i.e. predicted to inhibit differentiation) small molecules were pooled, averaged and ranked according to the combined *p* values/Z-scores using the R package ggplot2. IPSC-NPC (refered here as IPSC-derived NSCs (iNSCs)) data were downloaded from the L1000FWD resource and tsne coordinates used to construct a geom dot plot via ggplot2, illustrating differences in the transcriptional impact of exposure to the selected small molecules. The R code provided in the resource (https://amp.pharm.mssm.edu/l1000fwd/api_page) was adapted for extracting small molecule target genes in the positive and negative ranges (i.e. increased or decreased upon drug stimulation) among those relevant to the OL lineage/input expression profiles. The target genesfor the lower concentration of LY294002 (3.3 µm and 0.37 µm) and the higher concentrations (10 µm, 3 datasets), were merged. whereas target genes for Triciribine were derived from the one available dataset. Next, target genes were processed for pathway analysis using an R interface of the webtool Enrichr [22] (https://cran.r-project.org/web/packages/enrichR/), modified to derive pathways from the extracted small molecule target gene lists and visualised using via dotplots using ggplot2 package. The upregulated pathways were maintained in the positive ranges of the combined scoring of *p* values/Z-score, whilst for downregulated pathways, values were converted to negative ranges. Pathway terms were shortened to fit within plots and their entire listings, together with raw transcriptomic datasets and output files used in this manuscript will be placed in github (https://github.com/kasumaz/LINCs-Pharmacogenomics) upon acceptance.

### 2.3 *In vivo* procedures and study approval

All animal handling and experimental procedures were conducted in agreement with institutional and ARRIVE guidelines and the Home Office Animals Scientific Procedures Act (1986) and following approval by the local relevant committees. Animals were housed under *ad libitum* feeding and maintained on a 12 h light/dark cycle condition. Pups were weaned at Postnatal day (P)21 and group housed until approximately P35–40, at which time they were individually housed. All experimental design, performance and analysis were conducted adopting guidelines for rigour and reproducibility in science [23]. Group sizes were determined *a priori*, based on power calculation of similarly conducted studies [2], [13], [21]. A sample size of n = 6 biological replicates was used for each age group and animals were randomly assigned to experimental groups. All experiments were conducted in triplicate and no samples were excluded. All outcome measurements were subsequently conducted blindly. Experiments were performed on the wildtype strain C57/BL6 and on transgenic mouse lines in which fluorescent reporters DsRed or the enhanced green fluorescent protein (EGFP) are under control of the oligodendroglial-specific promoters: proteolipid protein 1 (PLP) or Sox10 respectively as characterised previously [24]. Unless stated, all materials were purchased from Sigma-Aldrich. *In vivo* experiments were performed on mice aged between postnatal day P8 and P11. Mice were either perfused or killed humanely by cervical dislocation and brains removed rapidly and submerged into ice-cold fixative. Mice aged P8 were treated by intraventricular injections into the lateral ventricle daily for 3 days, and brains sampled at P11 following the final injection. Concentration of injected small molecules into the lateral ventricle were calculated and corrected based on previous spectrophotometry of a GSK3β inhibitor’s bioavailability over time which decreases by 20-folds [25]. Mice were deeply anaesthetised with isofluorane and differing concentrations of LY294002 (Sigma-Aldrich), dissolved in sterile DMSO, sterile-filtered and co-administered with sterile saline were delivered into the cerebrospinal fluid (CSF) of the lateral ventricle using a Hamilton syringe, at a point 2 mm from the midline along the Bregma, and to a depth of 2 mm. EdU (5-ethynyl-2′-deoxyuridine) at 50 mg/kg was given by i.p. injection for two days during the first two days of small molecule treatment for aiding detection of proliferating cells and performed following manufacturers guidelines using Click-it EdU Alexa Fluor 555 imaging kit (Invitrogen).

### 2.4 Immunohistochemistry

Brains were immersion-fixed in 4% paraformaldehyde (PFA) in phosphate buffered saline (PBS), either for 3 h at room temperature (RT), or overnight at 4 °C. Following fixation, brains were washed in phosphate-buffered saline (PBS) and 50 µm thick coronal sections were serially collected using a vibratome [24]. Following washes in PBS, a blocking and permeabilization step was performed by incubating for 2 h at RT or overnight at 4 °C in 10% normal goat serum (NGS; Biosera) in 0.3% triton-X-100 in phosphate buffered saline (PBST). Sections were then incubated for 3 h at RT with agitation, or overnight at 4 °C, in primary antibodies diluted in NGS: rabbit anti-PDGFRα (1:400, gift from Prof Stallcup); goat anti-PDGFRα (1:200, R&D Systems); mouse anti-APC; CC1(1:300, Millipore); rabbit anti-GFAP (1:300, DAKO); mouse anti-PCNA (1:400, Sigma-Aldrich) mouse anti-Ascl1 (1:200, BD Biosciences); rabbit anti-Olig2 (1:400, Millipore); Goat anti-Mcm2 (1:300; Santa Cruz); Mouse anti-GFAP (1:500, Millipore; MAB3402). After washes in PBST, sections were incubated for 2 h at RT or overnight at 4 °C in the dark with the appropriate secondary antibodies conjugated with Alexafluor 488, 568 or 405 (1:500, Molecular Probes). Primary antibodies of different origin were diluted together in blocking buffer and co-dilutions of the appropriate secondary antibodies were used. Control experiments were performed using appropriate blocking peptides, where available, or otherwise by omission of the primary antibody. For PCNA, antigen retrieval was performed by pre-treating sections with PBST and NP-40 1% for 20 min to permeabilize the sections, and following brief washes in PBS, sections were immersed in pre-boiled citric acid and heated in a commercial microwave pressure cooker at full power for 30 s for 2 cycles. After final washes in PBS, tissue was mounted on poly-lysine-coated glass slides with Vectashield mounting medium (Vector Laboratories) and sealed with coverslips. Images were acquired using an LSM 5 Pascal Axioskop2 or LSM 710 meta confocal microscope (Zeiss). Fluorescence was visualised at 488 nm (green), 568 nm (red) and 405 nm (blue) using argon, HeNe1 and diode lasers respectively, using an x40 oil immersion lens with high numerical aperture (1.3 nm). Optic nerves were immersion-fixed in 4% PFA in PBS for 1 h at RT and following washes in PBS, whole-mounted on microscope slides in VectaShield (VectorLabs).

### 2.5 Adult and postnatal optic nerve tissue and organotypic cerebellar slice cultures

Organotypic cultures of mouse optic nerves were performed as described previously [13] and cerebellar slice cultures were prepared using tissue isolated from mice aged postnatal day P10–12 as previously described [26]. Briefly, a sample size of n = 6 biological replicates were used for the *ex-vivo* procedures which allowed us to obtain statistical significance and accounted for variability. Estimation of sample size was achieved by T and Z statistics using power calculation tools based on the analysis of previous studies [2], [13], [21]. Optic nerves aged P35–45 were removed with the retina intact were placed immediately in ice-chilled oxygenated artificial (a)CSF composed of: NaCl 133 mM, KCl 3 mM, CaCl2 1.5 mM, NaH2PO4 1.2 mM, HEPES buffer 10 mM pH 7.3, 0.5% penicillin and streptomycin (Invitrogen). For optic nerves, n = 6 optic nerves from 3 mice were used per experimental group for confocal microscopy analysis, and 12 nerves from 6 mice were used for transcriptomic analysis, according to power calculations hence ensuring sample sizes were adequate to detect statistical differences. Isolated optic nerve tissue was carefully cleaned of the arachnoid membrane and any attached peripheral/CNS tissue, then washed in aCSF and placed on semiporous culture membrane inserts (Millipore 0.4 µm). The medium (1 mL) for maintaining optic nerves consisted of 25% horse serum, 49% OptiMEM, 25% Hanks’s balanced salt solution, 0.5% 25 mM glucose, 0.5% penicillin and streptomycin. For preparing cerebellar slices, brain was rapidly removed and placed in oxygenated ice cold slicing solution containing (in mM) (25.95 NaHCO3, 1.39 NaH2PO4, 10 glucose, 124 NaCl, 2.95 KCl, 10 MgCl2, 2 CaCl2, 1 MgSO4, 1000 units/mL penicillin/streptomycin) and 300 µm parasagittal slices were cut using a vibrating microtome 5100 mz (Campden Instruments LTD). Slices were then analysed under the dissecting microscope to ensure maintenance of normal cytoarchitecture. Cerebellar slices were then transferred onto culture membrane inserts (Millipore 0.4 µm) and incubated in a medium that comprised 50% MEM (Eagle) with Glutamax-1, 25% EBSS, 25% horse serum, 130 mM glucose and 1% penicillin-streptomycin (all from GIBCO/Invitrogen). Optic nerves and cerebellar slices were maintained *ex vivo* at 37 °C in 95%O2/5% CO2 for 3 or 7 days respectively. LY294002 was added directly to the culture medium using the concentrations and duration as stated in the main text and vehicle DMSO used as control. LY294002 optimal concentration (L-LY294) for postnatal optic nerves (P8) and for cerebellar slices was set at 0.5 µM and 30 µM in adult optic nerves (P35–45). After 3 days for the optic nerve or 7 days for cerebellar slices, tissue was either fixed and prepared for confocal imaging, or processed for RNA extraction.

### 2.6 Cell counts, confocal microscopy and image analysis

Periventricular sections containing the lateral ventricle were analysed (> 3 sections per brain) using homogenous quantification procedures for NSCs [27]; counts of myelinating oligodendrocyte (MOL), newly formed oligodendrocyte (NFOL), OPC, TAP and GFAP+ astrocyte numbers in untreated controls confirmed that there were no significant differences between sections taken in this area [25]. Coronal brain sections were used throughout and cell counts performed in the dorsal SVZ, corpus callosum and cerebral cortex on orthogonally projected confocal z-stacks, of 230 µm x 230 µm in the x-y-plane, and 30 µm in the z-plane. 1 hemisphere was used for quantification and the other hemisphere for capturing representative images. For extracellular markers, a nuclear counterstain (DAPI (Invitrogen)) or Propidium Iodide (Sigma-Aldrich) was applied to aid quantification. Images were captured using a Zeiss LSM Meta 5.1 or Zeiss LSM 7.1 meta confocal microscope and processed with the latest Zeiss ZEN software (black edition), maintaining the acquisition parameters constant to allow comparison between samples. Measurement of a myelin index was performed on sections from PLP1-DsRed mice or Sox10-EGFP mice, together with MBP [24], providing a reliable readout of myelinated sheaths in the corpus callosum through a z-plane. The Zeiss ZEN software was used to determine the number of myelin sheaths crossing a diagonal transect was counted in each confocal z-section at 1, 5, 10, 15, 20, 25, and 30 lm (captured using a 403 objective). The resulting myelin index (MI) represents the density of DsRed+ myelin sheaths within constant volume as done for cell counts. DsRed+ OL processes were quantified by sampling randomly approximately 25 cells per brain section of each replicate animal comprising the medial corpus callosum. Images were captured with a x40 objectives in higher resolution and 1.5x zooming. Only fully visible DsRed+ cell bodies were quantified in this analysis and processes lengths of at least 10 µm. OL differentiation in the cortex was performed by quantification of APC+ OL soma’s and the extent of MBP+ processes in at least 30–40 µms in the z-plane (1 µm intervals). OLs with less than 10 thin/faint MBP+ processes were classified as NFOLs/MBPlow, whereas MOLs were determined by denser MBP immunolabelling on over 20 branched processes as “MBP+”. For analysis of optic nerve *ex vivo* cultures, each nerve was counted as a single sample and the total number of cells was counted midway along the length of the optic nerve, comprising a constant volume of 200 µm × 200 µm in the x–y-plane and 25 µm in the z-plane, commencing 15 µm below the pial surface. For all comparisons, the significance level was set to 5%; due to the explorative nature of this study, no adjustment was made to the significance level. Cell counts are presented as volumetric densities throughout, except for dorsal NSCs which have been presented as the averaged numbers per ventricle [27]. GFAP together with EdU were used to assess dorsal NSCs reliably, since markers used to assess NSCs in the adult are broadly expressed across NSC and progenitors during early postnatal development [19]. For optic nerves, there were 6 obtained from 3 mice in each experimental group and statistical analysis was performed as follows. Confocal micrographs captured with an x40 objective from every 5 confocal section in a series of 30 (n = 7 sections of 30 µm thickness) were analysed. Statistical analysis was conducted using GraphPad Prism v6: for multiple variables, using either Dunnett’s Multiple Comparisons test, or one-way analysis of variance (ANOVA) followed by Bonferroni’s posthoc test, and for two variable using unpaired *t*-tests (referred to as *t*-test) was applied.

### 2.7 Corpus callosum and cerebellar slice qPCR

The corpus callosum was microdissected using previously published protocols [28]. In brief, pups aged at P8 were treated as described above for 3 days with L-LY294 and 90 min following the final treatment were killed humanely by cervical dislocation and brains were rapidly dissected and placed in ice-cold postnatal specific coronal brain matrix (Zivic Instruments, Pittsburgh, PA, www.zivicinstruments.com). Tissue slices of 500 µm thickness were isolated and corpus callosum was microdissected for subsequent analysis by real-time quantitative polymerase chain reaction (qPCR). Two mice were pooled for RNA extraction for 1 “n” value. For the cerebellar slices maintained *ex vivo*, tissue was homogenised using Trizol (Qiagen) according to manufacturer’s protocol. RNA was extracted using the RNeasy microkit from Qiagen according to the manufacturer’s protocol. RNA quality and quantity was assessed by bioanalyzer (Agilent Technologies). Total RNA was converted into double-stranded cDNA using a cDNA synthesis kit (Superscript; Life Technologies, Carlsbad, CA). For rt-qPCR, amplified cDNA was loaded with 5 μM of forward and reverse primers, SYBR green mastermix, and DNAase/RNase-free H2O onto 96-well plates for Lightcycler 480 (Roche Diagnostics, Rotkreuz, Switzerland, www.roche-diagnostics.com). Relative gene expression was determined using the ΔΔ−ct method versus the housekeeping gene glyceraldehyde-3-phosphate dehydrogenase (GAPDH). Gene expression data are presented as mean ± standard error of the mean (SEM), and samples compared for significance using unpaired *t*-test (*t*-test) in GraphPad Prism v6. The following primers were designed using Primer Express 1.5 software and synthesised by Eurofins (Schönenwerd, Switzerland). Primers used:

*Aldh1a1*: ’5′CAGTAAACCTCCTGGCCAAA’3, 3′CCCTGTTTTCCCTACTTCCC5′

*Apc(Cc1):* 5'ATGAGTTTAAGGACGGCGGCGAAG3', 5'GTTCCAATGACCTTCCACCT3'

*Bax: 5′AGGATGCGTCCACCAAGAAGCT3', 5′TCCGTGTCCACGTCAGCAATCA3′*

*Bcl2: 5′CCTGTGGATGACTGAGTACCTG3', 5′AGCCAGGAGAAATCAAACAGAGG3′*

*Gapdh*: 5′GTGGAGTCATACTGGAACATGTGA’3, 3′AATGGTGAAGGTCGGTGTG5′

*Gfap*; 5′GCAGAAGCTCCAAGATGAAAC’3, 3′CCTTTCTCTCCAAATCCACAC5′

*Iba1: 5′TCTGCCGTCCAAACTTGAAGCC3', 5′CTCTTCAGCTCTAGGTGGGTCT3′*

*Mbp*: 5′ATTCACCGAGGAGAGGCTGGAA’3, 3′TGTGTGCTTGGAGTCTGTCACC5′

*Olig2*: 5′GACGATGGGCGACTAGACA’3, 3′CAGCGAGCACCTCAAATCTA5′

*Pcna*: 5′CAAGTGGAGAGCTTGGCAATGG’3, 3′GCAAACGTTAGGTGAACAGGCTC5′

*Pdgfra*: 5′AGAAAATCCGATACCCGGAG’3, 3′AGAGGAGGAGCTTGAGGGAG5′

*Sox10*: 5′*CTCAGCCTCCTCAATGAAGG3', 5′AGAAAGCTAGCCGACCAGTA3′*;

### 2.8 Whole genome transcriptome analysis

Complete details are provided in a recent study from the same authors, where identical procedures were followed for the preparation of RNA for Affymetrix GeneChip Mouse Genome 430 [13]. Downstream quality control steps and data analysis of produced .CEL image and .CHP image files were performed using Affymetrix GeneChip Operating Software. Agilent GeneSpring GX 12 software was used to normalise the datasets using the MAS-5 algorithm and further perform statistical analyses. GeneSpring was used to generate hierarchical clustering and the meta-analysis profiles of oligodendroglia-specific (OPC and MOL) signatures from published databases [29], [30]. Gene Ontology analysis was performed using ConsensusPathDB, String V10.5 and STITCH db, as described in detail previously [13]. In brief, the central nodes in STRING networks were selected by clustering nodes based on edge confidence score between 0 and 1 and in the analysis in Fig. 4 was set to a minimum interaction score at High Confidence: 0.70. Scores were computed based on several parameters (i.e. neighbourhood evidence, co-expression evidence, fusion evidence, co-occurrence evidence etc). High confidence interaction between two nodes is represented by thicker edges. Generated Microarray data are deposited in the NCBI gene expression omnibus (GEO) (http://www.ncbi.nlm.nih.gov/geo) and are accessible through GEO Series accession number GSE179189.

### 2.9 Bioplex immunoassay

Cerebellar slices of 300 µm thickness were isolated from P11 wild type mice C57BL/6 strain and incubated for 3 days as previously described above and elsewhere [26]. Thereafter, slices were washed in ice-cold cell wash buffer and lysed according to the manufacturer’s instructions. Total protein content was determined via BCA assay and samples and cell lysate controls were diluted to 10 μg/well (50 μL) in lysis buffer to ensure a constant sample input across all samples. Samples were treated according to manufacturer’s instructions. Briefly, blank samples (detection antibody) were loaded in duplicates in a 96-flat bottom well plate (Biorad) with previously diluted custom-made premixed fluorescent beads, which allowed the simultaneous quantification of phosphorylation levels in 7 analytes, and incubated for 15–18 h at RT under constant shaking. After washings in wash buffer, samples were incubated in detection antibody for 30 min washed again prior to incubation in Streptavidin-PE for 10 min. All incubations were performed at RT, under constant shaking and covered from light to protect the light-sensitive beads. Resuspended beads were then transferred to the Bio-plex Suspension Array system (BioRad) for quantification of phosphoproteins fluorescence intensity and results were expressed as mean fluorescence intensity (MFI). Further analysis was performed manually for excluding subtract blank MFI values (background) for each analyte from each sample. Statistical significance was calculated with GraphPad Prism 6.

## 3 Results

### 3.1 Pharmacogenomic screening for small molecules predicted to regulate myelination

**Fig. 1:**
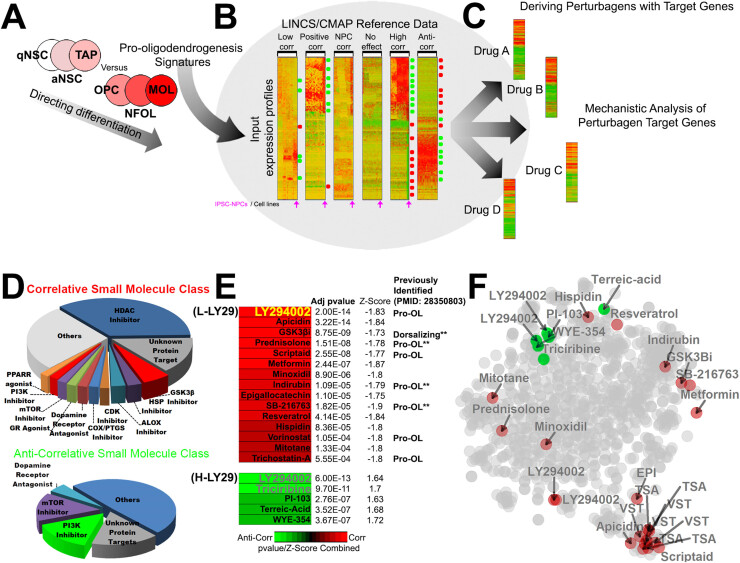
Querying the LINCS database for small molecules modulating oligodendroglia and insights into their cellular mechanisms of action. (A,B) Transcriptional signatures of early and late oligodendrocyte lineage stages are used to query the LINCS database, which contains drug-induced expression profiles for over 20,000 small molecules assayed in over 90 cells lines. To focus on the most OL-relevant data, only LINCS datasets comprising iPSCs-NSCs were queried. (C) Matching small molecule target genes are extracted for subsequent mechanistic investigations. (D) The broader mechanisms of action/target proteins of the highest ranked small molecules. (E) Heatmap output of the top ranking small molecules predicted to enhance (red) or inhibit (green) myelination, sorted by their adjusted (adj) *p* values, coloured by their combined pvalue/z-score (correlation in input profiles with the profiles induced by iPSCs-NSCs within the database). Small molecules tested are abbreviated in brackets. (F) tSNE plot illustrating the distance/similarities in target genes induced by top ranking oligodendroglial perturbing small molecules. Note the distance between LY294002 target genes (LY29) for the higher (green) and lower (red) concentrations. (For interpretation of the references to colour in this figure legend, the reader is referred to the web version of this article.)

Previously generated transcriptomic profiles for postnatal NSCs, adult NSCs and OL cells [2], [13], [18], [19], [21], were explored, to obtain essential landmark genes that characterise oligodendroglial differentiation (Fig. 1A). These signatures, which can be thought of as being pro- or anti-oligodendrogliogenic, were used to interrogate LINCS (https://clue.io) [11] and identify small molecules that shift the transcriptome of iNSCs to that of MOLs (Fig. 1B). Then, we analysed the small molecule target genes whose expression is significantly modulated in iNSC to either positively or negatively regulate OL differentiation (Fig. 1C, D). The predicted impact on many of these target gene networks fit known functions in OL, such as the pro-oligodendroglial effects of inhibiting GSK3β [25] and anti-oligodendroglial effects of inhibiting mTOR [31]. Others, such as the pro-oligodendroglial effect of HDAC inhibition, appear counterintuitive at first, based on existing knowledge of their role during OL lineage progression [32] (see also [33], [34] for the neuroprotective and OPC plasticity-promoting impact of transient HDAC inhibition). Significantly, inhibition of a number of pathways is predicted to have both pro- and anti-OL functions, most notably PI3K/Akt/mTOR inhibition (Fig. 1D). Consistent with this, the highest-ranking pro- and anti-oligodendroglial small molecule was LY294002, a potent inhibitor of PI3K/Akt signalling, with broad kinase-modulating activity depending on concentration and cellular context [35]. LY294002 exerts opposing transcriptional effects on iNSCs at low concentrations (2 µM, here termed L-LY29) and high concentrations (10 µM, here termed H-LY29) (Fig. 1E). The second highest-ranking anti-oligodendroglial small molecule was the specific Akt inhibitor Triciribine (TCN, Fig. 1E), and comparison of the target gene pathways and biological processes altered by the non-specific PI3K/Akt inhibitor LY294002 compared to the specific Akt inhibitor TCN provided insight into the potential mechanisms that determine the opposing effects of L- and H-LY29 on OL and myelination, with the “FAK-PI3K-mTOR pathway” being most prominent (Supplemental Fig. 1). In support of this, meta-analysis of the LINCS-derived target genes closely associated H-LY29 with TCN (Fig. 1F), consistent with the potential anti-oligodendroglial actions of H-LY29 being due to inhibition of PI3K/Akt/mTOR signalling, which is critical for OPC differentiation and myelination [36]. In contrast, the pro-oligodendroglial actions of L-LY29 are most closely linked to the effects exerted by GSK3β inhibitors and Metformin (Fig. 1F), both of which have been shown to rejuvenate OPC regenerative activity and promote remyelination [2], [9], [25], together with corticosteroids and the flavonoid epicatechin. Furthermore, STITCH protein-chemical network analysis predicted common mechanisms of action between LY294002 with previously identified pro-oligodendroglial small molecules such as Metformin, Prednisolone, Clemastine, Benztropine, Siponimod and Bexarotene (see Discussion below) (Supplemental Fig. 1C).

These findings suggested that all these agents are predicted to transduce via upstream target regulators of the PI3K/Akt signalling pathway, thus, exerting a modulatory regulation similar to L-LY29. Interestingly, L-LY29 is also closely associated with perturbation of HDACs (Fig. 1F), which has broad spectrum epigenetic effects that regulate OPC plasticity [34]. These analyses identified potential mechanisms that determine predicted opposing effects of H- and L-LY29 on oligodendrocytes and myelination.

### 3.2 Bipartite concentration-dependent effects of LY294002 on oligodendrocyte development *in vivo*

**Fig. 2:**
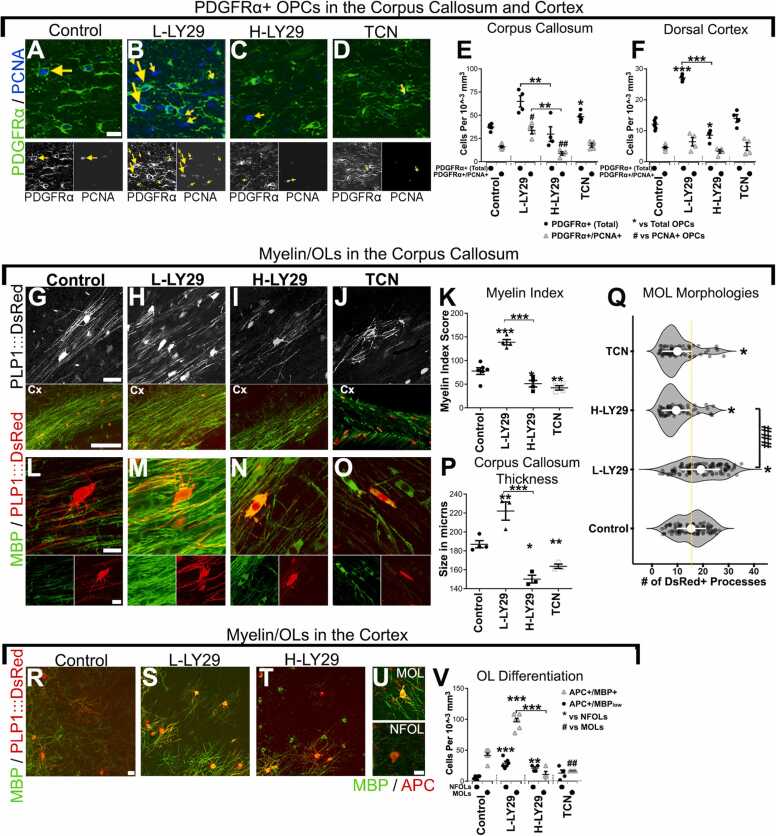
Concentration-dependent effects of LINCS-derived small molecules on oligodendroglia in the periventricular forebrain. P8 mice treated with saline/DMSO as controls, LY29 and TCN by infusion into the lateral ventricle for 3 days and analysed at P11. Immunostaining for PDGFRα for OPCs, PCNA for cells in S-phase and MBP for myelin. (A–F) Examination of OPCs in the indicated regions for their cell cycle states and quantifications in E, F. Arrows exemplify PDGFRα+/PCNA+ OPCs and small arrows are PDGFRα-/PCNA+ (pre-OPC). Images captured using x40 objective; scale bars  = 20 µm. (G–J) Top panels and lower panels respectively show single z-planes of PLP1-DsRed+ myelin sheaths and overviews (x40 objective) of the corpus callosum via MBP immunostainings. Scale bars = 20 µm in G or 150 µm. (L–O) Higher power cropped (x63 objective) confocal sections show the morphologies of OLs induced by treatment. Scale bar in L = 10 µm. Quantification of the myelin index in K, corpus callosum thickness in P or violin plots in Q illustrating the changes in DsRed+ OL phenotypes induced by the treatments. Each point represents the raw quantifications. (R–V) Examination of NFOLs and MOLs densities in the cortex where individual stages of OL units are resolvable as exemplified in V and quantified in W. E-T are maximum projections of confocal z-stacks of 10 µm thickness captured with a x20 objective and U captured by a 40x objective and are of 30 µm thickness. Scale bar = 20 µm in R and 10 µm in U. All histograms show the mean data quantifications + SEM in each region; n ≥ 4 animals and each n number represents 3–4 brain averaged per mouse;***/### p < 0.001,**/##, *p* < 0.01, */#, *p* < 0.05; *t*-test. Unpaired *t*-test used and Bonferroni’s posthoc test applied to reveal statistically significant differences between the two concentrations of LY-29 and controls.

The pharmacogenomic analysis identified LY294002 as a potential modulator of oligodendrogenesis and predicted differential effects of low and high concentrations. We tested this *in vivo* by directly injecting agents into the CSF of anesthetised mice for three days, commencing at postnatal day (P8) and analysing brains at P11, as described previously [25]. These ages correspond to the main period of oligodendrocyte differentiation and myelination in the corpus callosum and dorsal cortex, after which the numbers of OPCs decline sharply by half with a concomitant doubling in newly formed MOLs [24], [25]. The doses of small molecules injected were based on the previously calculated dilution effect of agents into the CSF, which is approximately 20-folds [25]. First, we determined the *in vivo* concentration-dependent effects of LY294002 and TCN (Supplemental Fig. 2); TCN was tested because it is a selective small molecule inhibitor of Akt, but does not inhibit PI3K, the direct upstream activator of Akt [37], [38], whereas LY294002 is a potent modulator of PI3K, the upstream activator of Akt, but has a broad kinase-inhibiting activity [35]. As predicted from the LINCS analysis, low doses of LY294002 (< 2 µM) and high doses of LY294002 (> 10 µM) had contrasting pro- and anti-oligodendroglial effects in the corpus callosum, whereas TCN was anti-oligodendroglial at all concentrations tested. Interestingly, the initial dose-response performed for LY294002 revealed a bell-shaped dose-response throughout in the corpus callosum and dorsal cortex (Supplemental Fig. 2). Upon confirming the optimal LINCS-guided doses, we performed a detailed *in vivo* analysis of the effects of 2 µM LY294002 (L-LY29) and 20 µM LY294002 (H-LY29), compared with 1.3 µM TCN on OPC, MOL and myelination (Fig. 2). Immunolabelling for PDGFRα and the cell proliferation marker PCNA demonstrated that L-LY29 increased the number of OPCs in cell cycle and doubled their number overall, in both the corpus callosum and dorsal cortex (Fig. 2A, B, E, F). In contrast, H-LY29 significantly reduced OPC numbers and proliferation (Fig. 2A, C, E, F), whereas TCN did not decrease OPC numbers (Fig. 2A, D, E, F), suggesting the negative effects of H-LY29 on OPCs are not mediated by PI3K/Akt signalling, but instead involve unknown mechanisms which remain to be elucidated.

Equivalent pro-oligodendroglial effects of L-LY29 were observed in MOLs, with a greater than doubling of the number of PLP+ MOLs in the corpus callosum, compared to controls (Fig. 2G, H; Supplemental Fig. 2A). The increased densities of MOLs were mirrored by an increase in the extent of MBP immunolabelling (Fig. 2L, M), quantified by the MBP+ myelin index, a measure of myelination (Fig. 2K), and by an increase in corpus callosum thickness (Fig. 2P). In contrast, both H-LY29 and TCN halved the numbers of DsRed+ MOLs when we treated a PLP1-reporter mouse (Fig. 2L, I, J; Supplemental Fig. 2A), and axonal myelination and corpus callosum thickness were markedly decreased (Fig. 2K, P). Overall, H-LY29 and TCN delays corpus callosum growth, given that from P8 to P11 it expands considerably (Azim and Butt 2011). These quantitative data indicate a large (350% in the corpus callosum and 500% in the cortex) increase in MOL densities in L-LY29 compared to H-LY29 or TCN (Supplemental Fig. 2A, B). In addition, MOLs appeared normal in L-LY29 compared to controls, but with increased numbers of DsRed+ oligodendrocytes and myelin sheaths (Fig. 2L, M), whereas in H-LY29 and TCN MOLs were atrophied exhibited abnormally oval somata (Fig. 2N, O). Quantification of DsRed+ processes (*p* < 0.05, ANOVA; ~25 cells counted per n number through a z-plane of 30 µM depth) revealed an approximate 20–25%increase in L-LY29 compared to controls (*p* < 0.05, ANOVA) and similar but opposite impact on the acquisition of complex MOL morphologies after treatment with H-LY29 and TCN (Fig. 2Q). As a further readout to assess the impact of the tested small molecules on the corpus callosum, qPCR was performed on microdissected tissue following the third day of intraventricular administration of L-LY294 as done previous [28] (Supplemental Fig. 2F). Whilst the oligodendroglial genes (*Olig2, Pdgfra, Mbp*) increased by over 2-folds compared to controls, those expressed selectively by astrocytes (*Gfap* and *Aldh1a1*) were significantly downregulated and no changes to *Iba1* gene which is expressed by microglia. In addition to *Pcna* expression increasing in the corpus callosum following L-LY294 (further shown above in Fig. 2A,B), the *Bcl2* and *Bax* genes were measured, which are readouts of survival and cell death, respectively. The results showed that whilst there were no changes in survival in the corpus callosum by L-LY294, cell death was significantly increased (Supplemental Fig. 2F). To validate the changes in reduced astrocyte-related transcripts, GFAP immunostaining was used to quantify astrocytes in the corpus callosum which was significantly reduced by approximately 20% (Supplemental Fig. 2G). In addition to the changes in cell survival/death as measured by qPCR, the number of dead (Propidium Iodide+) OL lineage cells was low and did not differ between treatment groups, ruling out cell stage-specific effects on viability as driving the observed shifts in lineage composition (Supplemental Fig. 2H). Follow-up studies are required to address further adverse effects induced by H-LY294 and TCN onto neighbouring cells in the corpus callosum, although the extent to which L-LY294 enhances oligodendrogenesis helps validate its novel effects as predicted by the LINCS analysis.

Next, OL differentiation was studied in the cortex since due to their lower density, individual terminally differentiated OLs are more clearly distinguished. OLs were evidently atrophied and supported far fewer myelin sheaths in H-LY29 compared to L-LY29 and controls (Fig. 2R–T). Immunolabelling for APC and MBP, which are expressed sequentially in differentiating oligodendrocytes, showed L-LY29 significantly increased the density of both APC+/MBPlow (0–10 branching processes) (NFOLs) and APC+/MBP+ MOL (> 20 branching processes) (Fig. 2U, V), whereas the main effect of H-LY29 and TCN was to impair the differentiation of APC+/MBP+ MOLs (Fig. 2V), consistent with evidence that Akt is required at the onset of OL terminal differentiation [39]. The results validated the pharmacogenomic analysis and demonstrated that LY294002 exerts a bipartite concentration-dependent effect on OL lineage cells, with high LY294002 having an anti-oligodendroglial action comparable to inhibition of PI3K/Akt signalling by TCN, whereas low LY294002 massively increased the generation of OPCs and promoted differentiation of MOLs by unresolved mechanisms.

### 3.3 LY294002 and TCN modifies oligodendrocyte generation from NSC of the dorsal SVZ

**Fig. 3:**
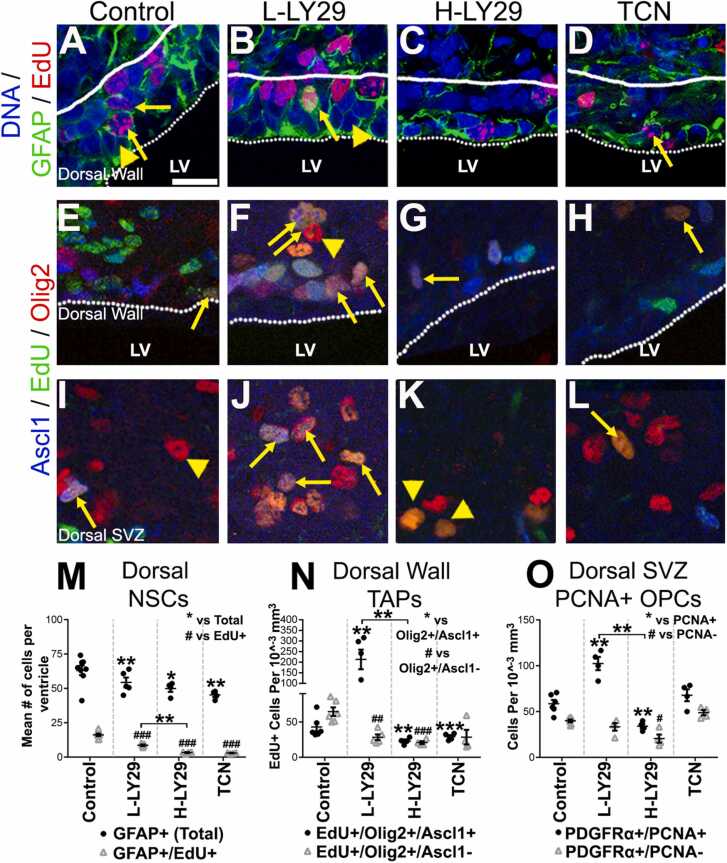
LINCS-derived small molecules differentially modulate oligodendrogenesis. P8 wildtype mice were treated with control (saline/DMSO), LY29 or TCN by infusion into the lateral ventricle for 3 days and periventricular/SVZ tissue analysed at P11. EdU was given at P9 and P10 by i.p. injections to aid lineage progression/proliferative cell quantifications in the SVZ. Scale bar = 20 µms. (A–D) GFAP (and staining for EdU for cells that cycled during treatment) for identification of NSCs adjacent to the dorsal ventricular wall. Arrows and arrowheads exemplify NSCs which cycled during treatment and remained quiescent, respectively. Scale bar = 20 µms for all micrographs, captured using a x40 objective. (E–L) Ascl1 and Olig2, identifying TAPs and oligodendrocyte lineage cells, respectively. Cells were examined directly close to the dorsal wall in E and within a 70 µm space between the ependymal layer and developing corpus callosum. Arrows and arrowheads exemplify TAPs committed to the oligodendrocytes and OPCs, respectively (Ascl1-/EdU+/Olig2+). (M–O) Quantification of dorsal NSCs (M), TAPs (N) and OPCs (O) are expressed as mean ± SEM in each region; n ≥ 4 animals; quantification performed on at least 3 brain sections and 3 regions of interest per brain section. ***/### p < 0.001, **/##, p < 0.01, */#, p < 0.05; *t*-test. Unpaired *t*-test used and Bonferroni’s posthoc test applied to reveal statistically significant differences between the two concentrations of LY29 and controls.

The results above demonstrated that LY294002 alters the development of forebrain MOLs, which are generated from OPCs that migrate from the dorsal SVZ and are derived from spatially defined pools of NSCs [3], [40]. We therefore examined the effects of LY294002 on NSC and oligodendrogenesis in the dorsal SVZ, as characterised previously [28], [40]. NSCs were identified as GFAP+ cells with a radial morphology adjacent to the ventricular surface, either as proliferating (GFAP+/EdU+) or non-proliferating (GFAP+/EdU-) NSCs (Fig. 3A–D). In the ages sampled (P11), GFAP expression is relatively specific to dorsal NSCs, whereas a range of other markers are expressed more broadly in SVZ progenitors. Newly generated pre-OPCs/TAPs (transiently amplifying progenitors) were identified by their co-expression of Ascl1 and Olig2 (Olig2+/Ascl1+), whilst OPCs were identified as Olig2+/Ascl1- (Fig. 3E–L).

Quantification demonstrated that GFAP+/EdU+ and GFAP+/EdU- NSCs were significantly decreased by all three treatments (Fig. 3M), but in addition H-LY29 and TCN treatments reduced GFAP expression in NSCs and almost completely abolished their proliferation (Fig. 3C, D). In contrast, newly formed oligodendroglial lineage cells exhibited differential responses to treatments compared to controls (Fig. 3E, I, N), whereby TAPs (Olig2+/Ascl1+) and OPCs (Olig2+/Ascl1-) were markedly increased by L-LY29 (Fig. 3F, J, N). However, these two populations were significantly reduced by H-LY29 (Fig. 3G, K, N), whilst TCN significantly decreased TAPs (Fig. 3H, L, N), but had no significant effect on OPC generation (Fig. 3L, O). In addition, quantification of definitive OPC pools in the dorsal SVZ, using immunostaining for PDGFRα and PCNA (as illustrated in Fig. 2A–F), demonstrated that their population is more than doubled by L-LY29, significantly reduced in H-LY29 and not significantly altered by TCN (Fig. 3O). Overall, these data indicated that L-LY29 drove the generation of oligodendroglial cells from NSCs of the dorsal SVZ and promoted the expansion of newly formed OPC, whereas H-LY29 and TCN almost completely ablated oligodendrogenesis.

### 3.4 LY294002 regulates oligodendrocyte generation in developing white matter ex vivo

Our results demonstrated that LY294002 regulates the generation of OL from the dorsal SVZ. Importantly, endogenous OPCs are a further major source of MOLs in the developing and adult brain and are potential targets of small molecules [25], [41]. The optic nerve is an excellent model for systems biological analysis of the mechanisms of action of small molecules on glial cells, since it does not contain neuronal nuclei and mRNA transcripts isolated from the optic nerves are glial, with insignificant levels from other cell types, such as endothelium [13], [42]. See also Supplemental Fig. 3,B for timeline of *ex vivo* experiments performed. To distinguish between the effects of LY294002 on NSCs and endogenous OPCs, we therefore examined the effects of LY294002 *ex vivo* on first the postnatal (P8) optic nerve as well as in the adult optic nerve (P35–45; see below), a typical white matter tract that contains endogenous OPCs, but not NSCs [13], [25], together with cerebellar slices from P10–12 Sox10:EGFP reporter mice to identify all oligodendroglial cells (OPC/MOL), as described previously (*13, 34*). In the postnatal optic nerve, LY294002 displayed strict dose-dependent effects, with L-LY29 increasing Sox10+ cells and H-LY29 almost completely ablating oligodendrocytes (Supplemental Fig. 3C–F); the pro-oligodendroglial effects of L-LY29 were confirmed in cerebellar slices, via both histology and qPCR-based analysis of oligodendroglial transcripts, confirming a marked increase in *Sox10* and *Mbp* (Supplemental Fig. 3G–J). In the optic nerve and cerebellar slices, OPCs are the sole source of newly generated OLs and these results verify that L- and H-LY29 have a bipartite effect on endogenous OPCs, as observed in the forebrain and predicted by pharmacogenomics.

### 3.5 Transcriptomic profiling of LY294002-responsive signalling pathways that regulate oligodendroglial lineage progression in adult optic nerves ex vivo

**Fig. 4:**
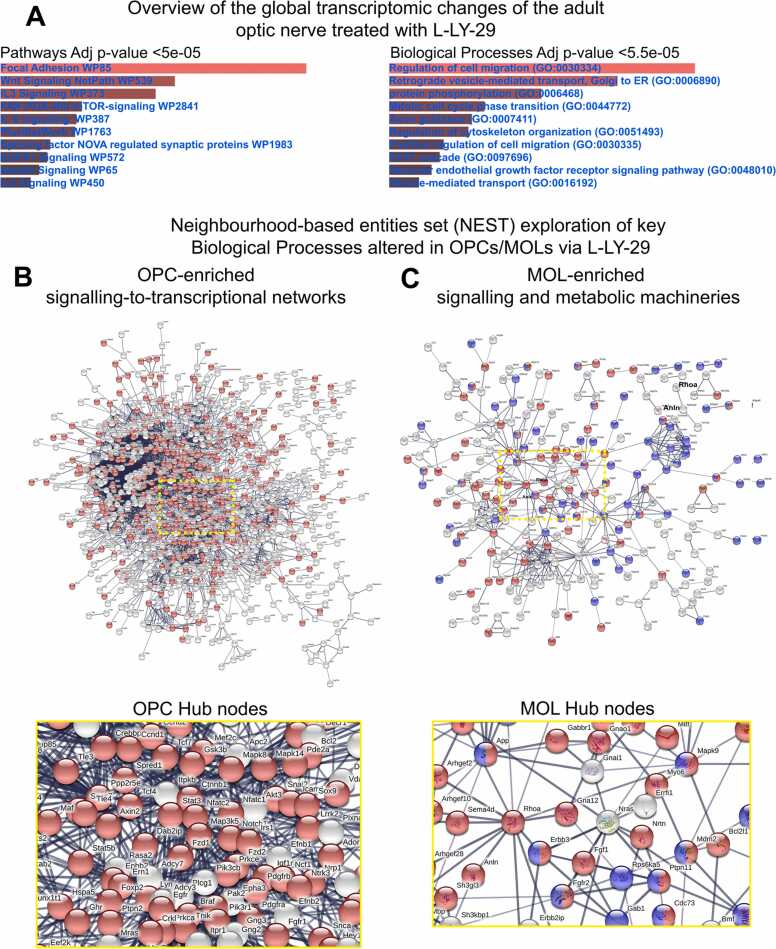
Defining the L-LY29 induced genes in the adult optic nerve and resolving OPC/MOL cellular networks. (A) Significantly differentially expressed transcripts induced by L-LY29 in the adult optic nerve (see also Fig. S4H) were inspected for signalling pathways and BP alterations, using the webtool Enrichr. The top 10 are presented and ranked by their adjusted (Adj) *p*-values. (B, C) Neighbourhood-based entities set analysis (NEST) of OPC- and MOL-enriched genes identified by STRING networks for predicted protein–protein interactions (circled in red). Central nodes were cropped and expanded to highlight the essential upstream factors regulated by L-LY29 and nodes are coloured as per key Biological Processes (see corresponding results section for the Biological Processes represented in coloured nodes). Central nodes in STRING V10.5 are selected by clustering nodes based on edge confidence score between 0 and 1 with a set minimum interaction score of 0.70. High confidence interaction between two nodes is represented by thicker edges. (For interpretation of the references to colour in this figure legend, the reader is referred to the web version of this article.)

We next used a combined neurobiological and transcriptomic analysis of adult mouse optic nerve to determine the effects of LY294002 on OL lineage cells (Fig. 4, Supplemental Fig. 4). First, LY294002 concentration was adjusted to take into account the developmental changes of the adult optic nerve such as increased length and diameter, maturation of myelination and developmental stage of the of the pia which at this age (P35–45) is fully mature and behaves as an impediment for drug permeability (see also the schematic overviews of the optic nerve in Supplemental Fig. 3A,B) [43]. Then, we verified that L- and H-LY29 retained the bipartite effect on OLs in the adult white matter as observed in postnatal models and *in vivo*, with L-LY29 (30 µM) significantly increasing both Sox10+ OPC/MOLs and PLP+/MOLs, whilst H-LY29 (50 µM) significantly decreased Sox10+ OPC/MOL, but not PLP+/MOL (Supplemental Fig. 4A–H). In contrast, the pro-oligodendroglial effects of L-LY29 are at odds with it acting via PI3K/Akt signalling. To resolve this, we performed a differential transcriptomic analysis of young adult optic nerves treated with L-LY29, compared to controls or H-LY29 (Supplemental Fig. 4I–L). Consistent with the striking bipartite effects of L- and H-LY29, only a relatively small number of genes (85) were common to both treatment groups (Supplemental Fig. 4K; Table S1), and STRING and GO analysis highlighted networks and biological processes associated with development as being common to L- and H-LY29, with *Igf1* representing a common core hallmark (Supplemental Fig. 4M). In comparison, differential analysis identified the genes that were regulated by L-LY29 (Supplemental Fig. 4J) and, using the webtool Enrichr, the key L-LY29-responsive gene pathways were identified as ‘Focal Adhesion’, ‘Wnt Signalling’ and ‘FAK-PI3K-mTOR signalling’, while Biological Processes induced by L-LY29 included ‘Regulation of cell migration’, ‘Retrograde vesicle-mediated transport’, ‘Protein phosphorylation’ and ‘Mitotic cell cycle phase transition’ (Fig. 4A). To elucidate the OL-specific L-LY29-responsive transcriptional networks, we interrogated our curated expression profiles of OPC- and MOL-enriched genes (Supplemental Fig. 4L; see Methods for details), which are visualised in a NESTED network for exploring the Biological Processes leading to pro-oligodendroglial effects of L-LY29 (Fig. 4B, C).

The most significant L-LY29-responsive OPC pathways are associated with cell cycle and differentiation, together with metabolism, nervous system development (*p* < 6.42e-46), neurogenesis (*p* < 1.07e-37) and cell morphogenesis (*p* < 1.97e-31). Critical L-LY29-responsive signalling mediators in OPCs are *Egfr* and *Fzd1/2,* with associated key transcriptional regulators *Stat3* and *Tcf4,* indicating key roles of EGFR and Wnt signalling in mediating the pronounced effects of L-LY29 on OPCs, consistent with published evidence (Fig. 4B) [25], [28], [40], [44], [45]. GO analysis of L-LY29-responsive MOL genes identified the most prominent biological processes as “cell differentiation” (Fig. 4C; Red, *p* < 3.68e-08; PPI< 1.54e-12) and cellular protein metabolic process (Fig. 4C; Blue, *p* < 2.55e-05), with central roles for *Rhoa* and *Anln* (Anillin), which have established roles in regulating the expression of major myelin proteins [46], [47]. Network analysis demonstrates that *Rhoa* is at the core of a number of key signalling networks (Fig. 4C; Red, *p* < 3.68e-08; PPI < 1.54e-12), including known pro-oligodendroglial mediators *Fgfr1* [24] and *Erbb3* [36]. The latter regulates OLs via RAF-MAPK and PI3K/Akt, both of which were identified by pharmacogenomics as key potential targets for controlling oligodendrogenesis (Supplemental Fig. 1) and are shown to be modified by LY294002 (Supplemental Fig. 2). These analyses identified stage-specific signalling pathways by which L-LY29 mediates the observed profound neurobiological effects on young adult OLs, with key roles for EGFR and Wnt signalling in massively expanding OPCs, and for FGFR1 and ERBB signalling in promoting myelination.

### 3.6 Validation of systems biology characterisation of cell-specific L-LY29-responsive signalling mechanisms

**Fig. 5:**
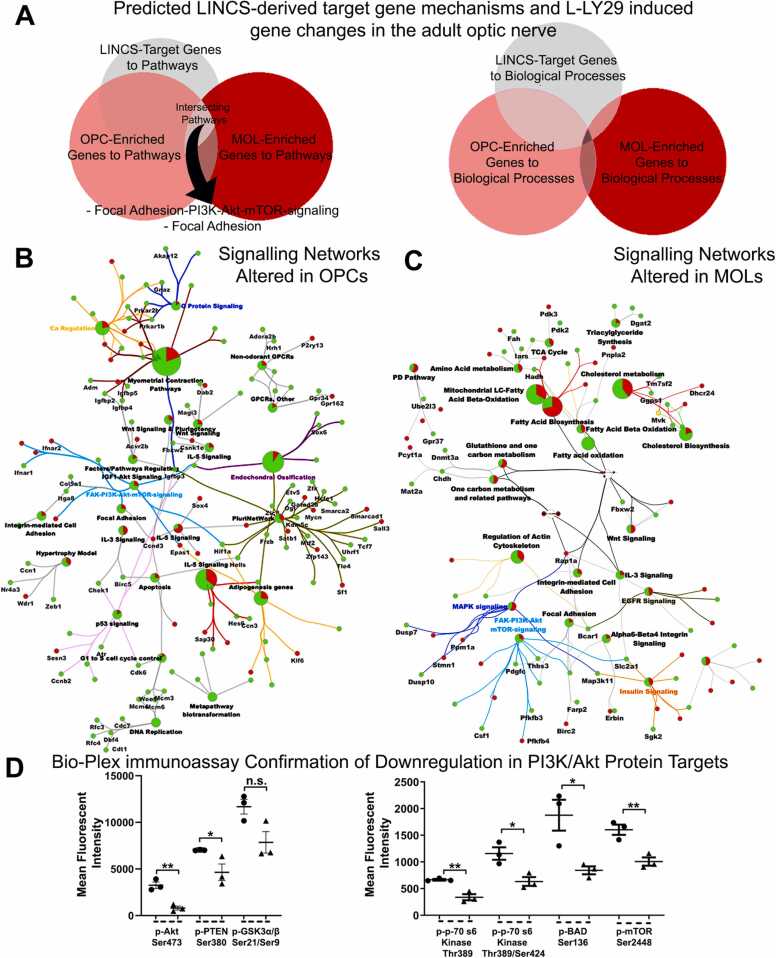
Validation and confirmation of signalling network alterations caused by L-LY29 by phosphoprotein immunoassay. (A) Comparison of LINCS-derived target genes processed by Enrichr to identify signalling pathway and BP alterations at two oligodendrocyte lineage stages (OPC-enriched profiles and MOL-enriched profiles) following L-LY29 treatment of the adult optic nerve. (B, C) Cluego signalling pathway networks of changes in defined stages of OL differentiation. Nodes in red or green represent those that are respectively upregulated or downregulated upon L-LY29. (D) Cerebellar slices from P11 mice were maintained in culture for 3DIV in control medium or medium containing 0.5 µM LY29 and phosphoproteins were assessed by Bio-Plex immunoassay. Data are mean ± SEM (n = 3 per group, one slice each from a different littermate) fluorescence intensity. phospho-Akt (Ser473), phospho-PTEN (Ser380) and phospho-GSKα/β (Ser21/Ser9); *p < 0.05, **p < 0.01, two-tailed unpaired *t*-test. (For interpretation of the references to colour in this figure legend, the reader is referred to the web version of this article.)

We next performed a validation of the cellular effects predicted by LINCS (Supplemental Fig. 1) compared to the cellular effects resolved experimentally (Fig. 4, Supplemental Fig. 4), by interrogating L-LY29-responsive oligodendroglial genes against the LINCS-derived target gene pathways and Biological Processes (Fig. 5A). Significantly, LINCS genes enriched in both L-LY29-responsive OPC and MOL genes were associated with the pathway ‘Focal adhesion-PI3K-Akt-mTOR-signalling’, which is a key target for pro-oligodendroglial small molecules (Supplemental Fig. 1). Next, we constructed cell-specific L-LY29-responsive signalling networks in adult OPCs and MOLs, using Cytoscape ClueGO (Fig. 5 B,C; see Methods for details). Importantly, the results confirmed that pathway terms Focal adhesion-PI3K-Akt-mTOR-signalling and focal adhesion” were both downregulated by L-LY29 in OPCs and MOLs (Fig. 5B, C). Focal adhesion signal transduction is complex and exerts opposing roles on OL maturation [48], whereas signalling networks emanating from PI3K/Akt and affecting signalling by PTEN, GSK3β and mTOR were particularly evident in the effects of L-LY29 on OPCs and are known to be inhibitory in OPCs [25], [49].

To test the predicted impact of L-LY29 on PI3K/Akt/mTOR signalling, we performed multiplex immunoassays on cerebellar slices treated with L-LY29, which significantly reduced phosphorylation of Akt, mTOR, Pten, p70S6, and Bad (Fig. 5D; *p* < 0.05). Thus, these analyses identified stage-specific signalling pathways by which L-LY29 mediates the observed profound neurobiological effects on OLs and comprehensively validate the LINCS-generated catalogue of small molecules that have the potential to promote oligodendrocyte generation and myelination.

## 4 Discussion

Connectivity mapping holds considerable potential in the search for new therapies to promote repair in multiple neuropathologies [2], [13], [21]. However, it is important to assess the potential for contrasting biological actions of drugs depending on the cellular context and their potential dose-dependent effects on intracellular regulatory pathways. Here, using next-generation drug connectivity mapping LINCS [11], we have identified small molecules that have marked dose-dependent effects on OLs in the CNS. Surprisingly, LINCS analysis identified the small molecule kinase inhibitor LY294002 as the highest-ranking agent predicted to have both pro- and anti-oligodendroglial effects, depending on dose, which we fully validated in multiple neurobiological *in vivo* and *ex vivo* models. Moreover, analysis of LY294002-responsive genes identified the cell-specific mechanisms of action of low and high doses of LY294002 on OL lineage cells. The results demonstrated that a combined drug connectivity mapping and neurobiological strategy is a promising and direct approach to identify small molecules and transcriptional networks that have the potential to promote regeneration and repair in the CNS.

Important studies in the field have utilised high-throughput compound libraries on purified oligodendroglial or iPSCs lines for obtaining novel pro-myelinating small molecules [50], [51], [52], [53], [54], [55], [56], which were further clinically validated using mouse models of demyelination [50], [51], [52], [56], and ultimately introduced in clinical practice for MS [57]. Although some of these small molecules are beneficial, their mechanisms of action in promoting remyelination remain to be better defined. Our analyses identified potential interrelations between some of these small molecules among which, low doses of LY294002, hence predicting an upstream modulation of PI3K/Akt pathway. For example, Clemastine and Siponimod have been described to activate the PI3K/Akt pathway, which leads to successful remyelination *in vivo* [58], [59]. Similarly, neuroprotection via Prednisolone, which was highly ranked in our own pharmacogenomics analysis (Fig. 1E), can be abrogated by high doses of LY294002, suggesting a key role for PI3K/Akt signalling in the promyelinating effects of Prednisolone [60]. Hence, pharmacogenomic identification of small molecules reduces reliance on extensive model-specific experimental screening, enabling the characterisation of a handful of promising agents using translationally relevant, but poorly scalable experimental systems. Another notable advantage of drug connectivity mapping is the opportunity it provides to identify small molecule target gene networks, and thus streamline the design of pre-clinical experiments. In the present study, after mapping transcriptional drug responses in iNSCs onto an oligodendroglial differentiation axis, our meta-analysis of LINCS data predicted the PI3K/Akt modulator LY294002 as having dose-dependent pro- or anti-oligodendroglial actions. High doses of LY294002 was predicted to cause the demise of OLs via signalling networks and biological processes, also regulated by the specific Akt inhibitor TCN, e.g. Notch signalling (Supplemental Fig. 1). As predicted by our connectivity mapping, both H-LY29 and TCN were confirmed to negatively affect OL lineage progression *in vivo* and *ex vivo*, in line with effects on PI3k/Akt/mTOR signalling [16], [61], [62]. In contrast, L-LY29 upregulated essential drivers of oligodendrogenesis and differentiation, including Wnt signalling [28], [40]. These findings were verified *in vivo* in the SVZ and H-LY29 and TCN were shown to perturb common signalling networks in the SVZ that control specification of OPC from NSC, and consistent with known roles for Akt signalling in modulating dorsal NSC/TAPs survival, proliferation, and self-renewal [63].

The dorsal SVZ niche during postnatal development expresses over 50 distinct signalling ligands, which are derived from multiple cellular sources within and in the vicinity of the dorsal SVZ, and affect the activity of multiple intracellular pathways, influencing cell fate choices such as survival, self-renewal and commitment to differentiate [2]. Interestingly, our *in vivo* data reveals complex effects of L-LY29 on multiple stages of oligodendroglial lineage commitment and progression. The observed depletion of proliferating NSCs, accompanied by a dramatic increase in their immediate oligodendrocyte-committed progeny (TAPs) during development, point to a rapid effect of L-LY29 to promote commitment and progression along the OL lineage, consistent with upregulation of cell cycle activity and Wnt signalling [28], [40], as predicted by the LINCs target gene analysis (Supplemental Fig. 1A). Future studies will define the transcriptional alterations induced in NSCs/TAPs by which L-LY29 specifies early OL lineage cells.

Importantly, our *ex vivo* data from both postnatal and adult optic nerves, together with cerebellar white matter tracts devoid of NSCs and TAPs supported our *in vivo* analyses and demonstrated that L-LY29 promotes OL generation from both the SVZ and endogenous OPCs by triggering a cascade of pro-oligodendrogenic signalling networks, consistent with its broad kinase-targeting activity and the pleiotropy of downstream effects of PI3K/Akt/mTOR signalling. LY294002 reversibly inhibits PI3K, but depending on the dose can also bind to numerous other proteins [35], such as the bromodomain proteins BRD2-4, which are expressed along the entire oligodendroglial lineage and have the potential to promote oligodendroglial differentiation [64], [65]. Intriguingly, alterations in GO terms “cell adhesion”, “PTEN signalling”, “Wnt signalling” and “cytoskeletal arrangements” are amongst the most significant transcriptional changes upon partial Brd protein blockade in NSCs [66]. These data are matched by the major terms predicted and confirmed by our validatory transcriptomic experiments following exposure of optic nerves to L-LY29, lending support to the possibility that targeting Brd proteins could represent a strategy for promoting myelin repair [67]. Thus, future studies will aim to assess whether the next generation of pan PI3K inhibitors at the lower concentrations when applied by direct infusion into the lateral ventricle, promotes oligodendrogenesis as in the present study.

PI3K signalling regulates a plethora of pathways that affect metabolism, growth and maturation of individual cells [68]. Current therapeutic strategies targeting the PI3K/Akt axis are hindered by its relevance across different biological systems. In addition, modulators and effectors of PI3K signalling, such as mTOR, RAC and S6K, are important for “fine-tuning” and tightly regulate signalling activity. For these reasons, different strategies are being explored to reach therapeutic efficacy which include moving from a continuous dose therapy to pulsed/intermittent dosing [68], to the exploration of effects elicited by low and high compound doses as we reported in our study. Interestingly, although LY294002 is considered a pan-class I PI3K antagonist, it exhibits concentration-dependent affinity to different isoforms, with an IC50s *in vitro* of 0.55 µM, 1.1 µM, 1.6 µM and 12 µM against p110α, β, δ and γ [69]. Thus, lower dosage of LY294002 might favour inhibition of p100α and δ, which may possess different regulatory properties in OLs. Future studies aim to characterise the role of different PI3K isoforms in OLs and the intriguing possibility of using selective antagonists that may favour the inhibition of p100α and δ. Furthermore, H-LY29 (and also TCN) are toxic at higher concentrations *in vivo*, it would be highly apparent that there would be numerous indirect effects via astrocytes, microglia and axons. For example, high concentrations of LY294002 have been shown to ablate the protective effect of astrocyte-derived media on OPCs *in vitro* [70]. Thus, it may be possible that toxicity induced via H-LY29 will be due to a combination of direct and indirect modes of regulation on OL lineage cells. Future studies should be aimed at dissecting the role of LY294002 on other cell types.

We predict that combining traditional drug discovery screening approaches with pharmacogenomically obtained small molecules will prove synergistic for translational medicine [50], [51], [52], [71], facilitating the identification of potentially effective small molecules and accelerating the dissection of their context-specific mechanisms of action. Future experiments performed in an *in vivo* model of demyelination will aim to resolve therapeutic strategies that may be successfully applied to promote oligodendrogenesis in an regenerative context, by exploiting pharmacogenomically predicted dose-dependent effects of a small molecule. Further studies are needed to assess the functional outcome of L-LY29-boosted oligodendrogenesis and to dissect the mechanisms by which it promotes dorsal NSC-to-oligodendroglial commitment and accelerated myelination and remyelination. These findings are of potential translational interest given the lack of effective drivers of OPC recruitment to demyelinated lesions in MS, which may leave residual OLs as the main source of newly deposited myelin during remyelination [72]. Given the realisation that adult oligodendroglia comprise a set of regionally and possibly functionally diverse populations [73], the strategy outlined in the present study may facilitate efforts for targeting molecularly defined oligodendroglial populations for myelin repair.

## 5 Conclusion

The extensive datasets hosted by the LINCS consortium, comprising gene expression profiles generated using a diverse set of target cells under a broad set of conditions, provide unparalleled access to the multidimensional interactions emerging when assessing drug-gene interactions at whole-genome levels. Using LINCS, we identified a range of small solutes that have the potential to regulate OLs by targeting diverse intracellular signalling pathways. We comprehensively validated our drug networking strategy in multiple *in vivo* and *ex vivo* postnatal and adult models, using diverse techniques to analyse the dose-dependent effects of LY294002, including lineage progression characterisation, transcriptomics and signalling pathway assays. However, conventional genetic approaches to target specific aspects of intracellular regulatory pathways often yield contradictory findings and cannot resolve dose-dependent effects, which are essential for the development of new therapies [31], [62], [74]. In contrast, our demonstration of drastically bipartite dose-dependent effects of the PI3K inhibitor LY294002 highlights the power of our pharmacological targeting approach to resolve complex cell-specific signalling networks that are otherwise not identifiable via conventional genetic strategies. These techniques offer unprecedented opportunities to gain insights into hard-to-predict context-specific mechanisms of action of small molecules to promote regeneration and repair in multiple neuropathologies.

## Funding

This work was supported by grants from the Biotechnology and Biological Sciences Research Council (BBSRC, UK) (BB/M029379/1), 10.13039/501100000265Medical Research Council (MRC, UK) (MR/P025811/1), Multiple Sclerosis Society of the UK (40), University of Portsmouth PhD Programme, MSCA Seal of Excellence @ UNIPD and NVIDIA Hardware Grant, German Research Council (AZ/115/1-1; AZ/115/1-3), Swiss National Funds (P300PA_171224).

## CRediT authorship contribution statement

**FP**: Conceptualization, Data curation, Formal analysis, Methodology, Supervision. **AR**: Conceptualization, Writing, Validation, Data curation, Data analysis, Funding acquisition. **GW**: Data curation, Analysis, Investigation, Validation, Methodology. **FC**: Writing, Validation, Data analysis. **AB**: Funding acquisition, bbs acquisition, Project administration, Writing, Methodology, Supervision, Validation. **KA**: Funding acquisition, Investigation, Data curation, Methodology, Project administration, Supervision, Validation, Writing.

## Data Availability

Data is available through data repository as described in the methods section

## Appendix A Supplementary material

.

Supplemental Fig. 1: **Prediction of the cellular effects of LINCS-derived small molecules on oligodendrocyte lineage cells.** Small molecule target genes Pathway and BP term memberships as revealed by querying (**A**) Wikipathways and (**B**) GO Biological Processes. Geom dot plots illustrating Pathway/BP terms were shortened to fit, arranged by their combined scorings (log*p*value/z-score) and point sizes reflect the relative enrichment. Full list of small molecule target genes, pathways and Biological Processes are available with the raw data. (**C**) Functional Protein-Chemical Network Analysis (STITCH) was performed to identify the potential common mechanisms between LY294002 and known pro-oligodendroglial small molecules Metformin, Prednisolone, Clemastine, Benztropine, Siponimod and Bexarotene. Network analysis predicts a modulation of PI3K/Akt signalling ( core node (KEGG database *p* < 8.4 e-08) upstream of LY294002. PPI enrichment *p* value < 0.0001

.

Supplemental Fig. 2: **Concentration-dependent effects of LY294002 (LY29) and Triciribine on stage-specific oligodendroglia in the corpus callosum and cortex.** Small molecules were infused at a range of concentrations into the lateral ventricle for 3 days commencing at P8 and the periventricular corpus callosum and cortex were examined at P11. (**A-E**) Abundance of PLP-DsRed-positive oligodendrocytes (**A, C**) and PDGFRα-positive OPCs in wildtype mice (**B, D**) in the specified periventricular tissues. Concentrations are given in **E** as injected and dilution-corrected, based on the measured 20-fold dilution in the CSF (see Methods). Values are expressed as a mean number of cells per studied brain region performed in duplicate regions of interest from each hemisphere, from at least n = 4 per treatment; error bars represent the SEM. * *p* < 0.05, * * *p* < 0.01 Dunnett’s Multiple Comparisons test. (**F**) Mice were treated with L-LY29 for 3 days and 90 mins following the final injection, mice were sacrificed for corpus callosum microdissection and qPCR of selected transcripts as shown. 3 n numbers were used throughout; * *p* < 0.05, * * *p* < 0.01, * ** *p* < 0.001. (**G**) L-LY29 was infused into the lateral ventricle for 3 days commencing at P8 and the periventricular corpus callosum examined at P11 for immunolabelling of GFAP for quantification of astrocytes. At least 4 numbers were used; * *p* < 0.05. Representative confocal images (captured via a x40 objective) and are flattened confocal z-sections of 12 µms. Scale bar = 50 µm. (**H**) The optimal concentrations of LY294002 and Triciribine in E were tested as above in the Sox10-EGFP+ mice, in which all cells of the OL lineage are labeled. Quantification of PI labelled necrotic cells of the OL lineage (Sox10-EGFP+/PI+) or neighbouring cells (Sox10-EGFP-/PI+) in the periventricular forebrain in the stated regions. Data are the mean data quantifications + SEM in each region; n ≥ 4 animals; quantification performed on at least 3 brain sections and 3 regions of interest per brain section. * ** /### *p* < 0.001, * */##, *p* < 0.01, * */#, *p* < 0.05; *t*-test. Unpaired *t*-test used. Confocal micrographs in G exemplify cells in the corpus callosum, where both viable and dead EGFP+ OLs are found at higher densities. Images are maximum-intensity projections of z-stacks (acquired with a x40 objective) of 6 µm thickness (scale bars = 25 µm). Arrows exemplify decaying EGFP+ OLs.

.

Supplemental Fig. 3: **LY294002 regulates oligodendroglial cells in the postnatal optic nerve and cerebellar slices ex vivo.** (**A-B**) Schematic overview of optic nerve growth from postnatal periods to adulthood and timelines used for assessing the impact of LY294002 on OL differentiation. (**C-F**) Representative confocal images of optic nerves from postnatal Sox10-EGFP mice maintained for 3DIV, in control medium (DMSO), and medium containing increasing concentrations of LY29. Confocal micrographs of the whole-mounted optic nerve illustrates concentration- dependent effects of LY29 in the adult optic nerve. (**F**) Histogram of mean ( ± SEM) cell counts per constant FOV (n = 3 for each group); * * *p* < 0.01, * ** *p* < 0.001 (comparing controls to treatment groups), ####*p* < 0.0001, (comparing LY-29 0.5 µM to higher LY29 concentrations), One-way ANOVA followed by Dunnett’s multiple comparisons test. (**G, H**) Representative confocal images (captured via a x20 objective) of cerebellar slices from postnatal Sox10-EGFP mice maintained for 7DIV in 0.5 µM LY29-containing medium; scale bars = 100 µm. (**I**) Histogram of mean ( ± SEM) cell counts per constant FOV (n = 5 for each group). * * *p* < 0.01, two-tailed unpaired *t*-test. (**J**) *Ex vivo* cerebellar slices were maintained in 0.5 µm LY29 or control vehicle (saline/DMSO) conditions for 3DIV and tissue harvested for processing for qPCR for oligodendroglial transcripts. Data are normalised to the housekeeping gene GAPDH (ΔCt) and expressed as mean 2-ΔCt ± SEM (n = 3 for each treatment group). * *p* < 0.05, two-tailed unpaired *t*-test.

.

Supplemental Fig. 4: **Concentration-dependent effects of LY29 on adult optic nerve oligodendroglia and whole genome profiling for revealing genes regulated by LY29.** (**A-C**) Representative confocal images (captured via a x20 objective) from Sox10-EGFP (top panels) and PLP-DsRed1 (lower panels) optic nerves maintained in culture for 3DIV in control (DMSO) (A), 30 µM LY29 (**B**), or 50 µM LY29 (**C**); scale bars = 20 µm. (**D**) Histogram of mean ( ± SEM) cell counts per constant FOV (n = 6 for each group), * ** *p* < 0.001, (comparing vehicle control to all treatments), ##*p* < 0.01, ####*p* < 0.0001 (comparing 30 µM LY29 to 50 µM), One-way ANOVA followed by Tukey’s post-hoc test. (**H, I**) Heatmap of transcriptional changes occurring in the adult optic nerves incubated in control medium and in 30 or 50 µM LY29. Red, green and black represent probes upregulated, downregulated or showing no change, respectively, and the most significant probes shown on a volcano plot in **I**. (**J**) Venn diagram’s of probes specific to or common between the concentrations of LY29 (top Venn’s), meta-analysis of OPC-enriched (middle Venn’s) or MYOL-enriched (bottom Venn’s) profiles using publically available OPC- and MYOL-derived datasets (see Materials and Methods). (**K, L**) Protein interactome networks generated using the STRING database of genes common to both LY29 concentrations or those specific to H-LY29.
