# Supplementary figures and images for: Drug connectivity mapping and functional analysis reveal therapeutic small molecules that differentially modulate myelination
Source: Biomed Pharmacother. 2022 Jan;145:None. doi: 10.1016/j.biopha.2021.112436 (PMC8664715; doi:10.1016/j.biopha.2021.112436)

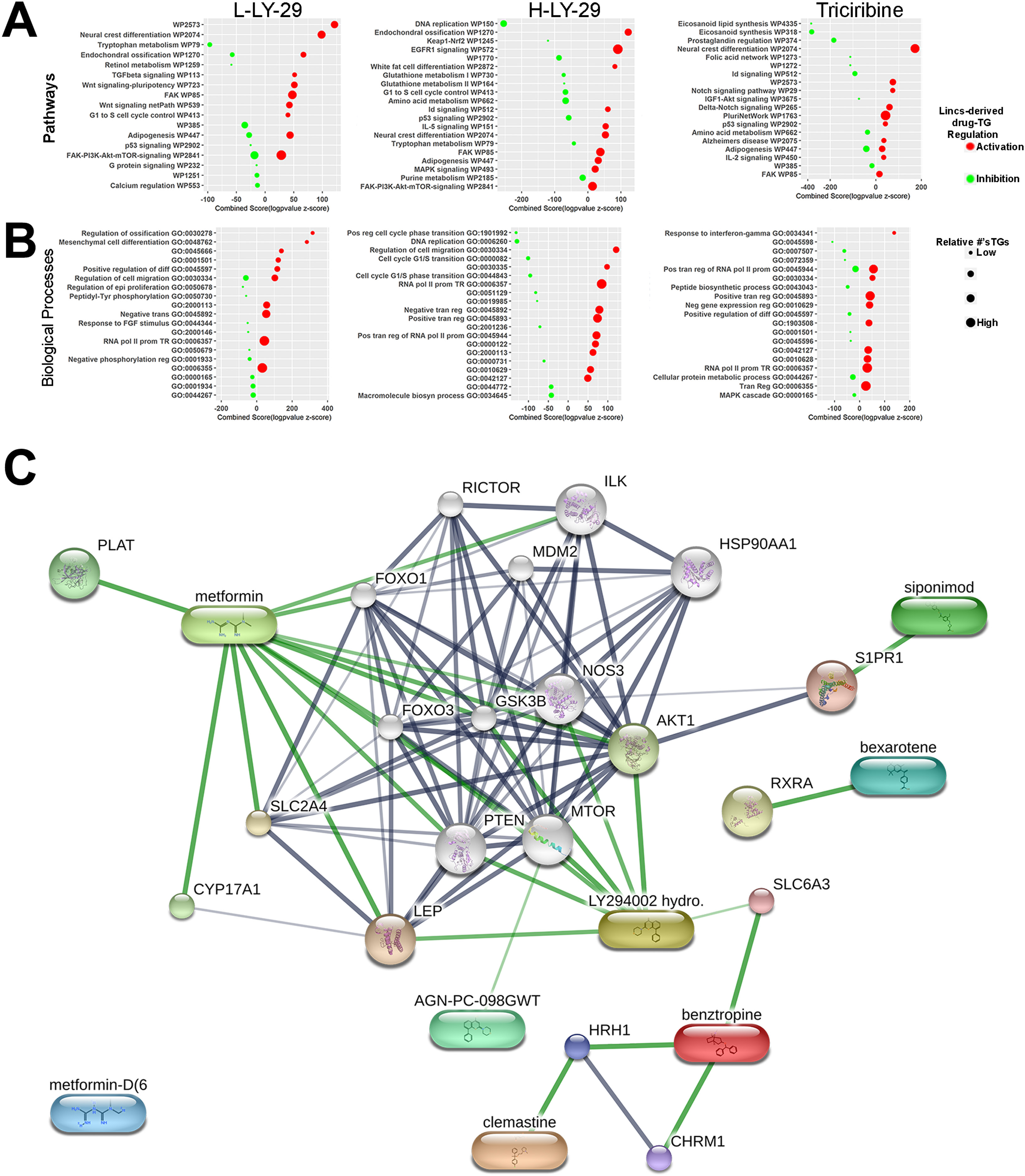

Supplement: Supplementary file 2 — Supplemental Fig. 1: Prediction of the cellular effects of LINCS-derived small molecules on oligodendrocyte lineage cells. Small molecule target genes Pathway and BP term memberships as revealed by querying (A) Wikipathways and (B) GO Biological Processes. Geom dot plots illustrating Pathway/BP terms were shortened to fit, arranged by their combined scorings (logpvalue/z-score) and point sizes reflect the relative enrichment. Full list of small molecule target genes, pathways and Biological Processes are available with the raw data. (C) Functional Protein-Chemical Network Analysis (STITCH) was performed to identify the potential common mechanisms between LY294002 and known pro-oligodendroglial small molecules Metformin, Prednisolone, Clemastine, Benztropine, Siponimod and Bexarotene. Network analysis predicts a modulation of PI3K/Akt signalling ( core node (KEGG database p < 8.4 e-08) upstream of LY294002. PPI enrichment p value < 0.0001 [file mmc2.jpg]

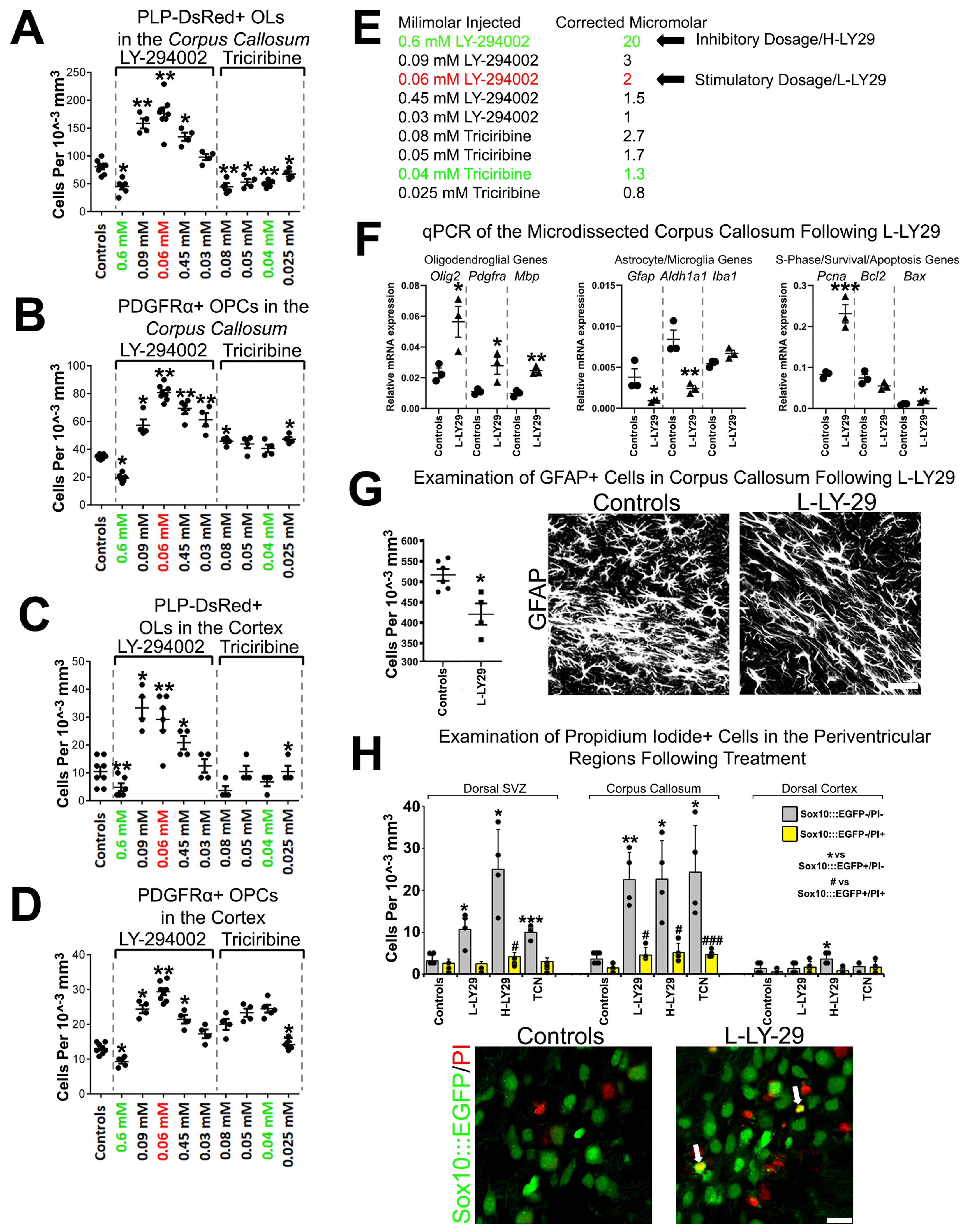

Supplement: Supplementary file 3 — Supplemental Fig. 2: Concentration-dependent effects of LY294002 (LY29) and Triciribine on stage-specific oligodendroglia in the corpus callosum and cortex. Small molecules were infused at a range of concentrations into the lateral ventricle for 3 days commencing at P8 and the periventricular corpus callosum and cortex were examined at P11. (A-E) Abundance of PLP-DsRed-positive oligodendrocytes (A, C) and PDGFRα-positive OPCs in wildtype mice (B, D) in the specified periventricular tissues. Concentrations are given in E as injected and dilution-corrected, based on the measured 20-fold dilution in the CSF (see Methods). Values are expressed as a mean number of cells per studied brain region performed in duplicate regions of interest from each hemisphere, from at least n = 4 per treatment; error bars represent the SEM. * p < 0.05, * * p < 0.01 Dunnett’s Multiple Comparisons test. (F) Mice were treated with L-LY29 for 3 days and 90 mins following the final injection, mice were sacrificed for corpus callosum microdissection and qPCR of selected transcripts as shown. 3 n numbers were used throughout; * p < 0.05, * * p < 0.01, * ** p < 0.001. (G) L-LY29 was infused into the lateral ventricle for 3 days commencing at P8 and the periventricular corpus callosum examined at P11 for immunolabelling of GFAP for quantification of astrocytes. At least 4 numbers were used; * p < 0.05. Representative confocal images (captured via a x40 objective) and are flattened confocal z-sections of 12 µms. Scale bar = 50 µm. (H) The optimal concentrations of LY294002 and Triciribine in E were tested as above in the Sox10-EGFP+ mice, in which all cells of the OL lineage are labeled. Quantification of PI labelled necrotic cells of the OL lineage (Sox10-EGFP+/PI+) or neighbouring cells (Sox10-EGFP-/PI+) in the periventricular forebrain in the stated regions. Data are the mean data quantifications + SEM in each region; n ≥ 4 animals; quantification performed on at least 3 brain sections and 3 regi [file mmc3.jpg]

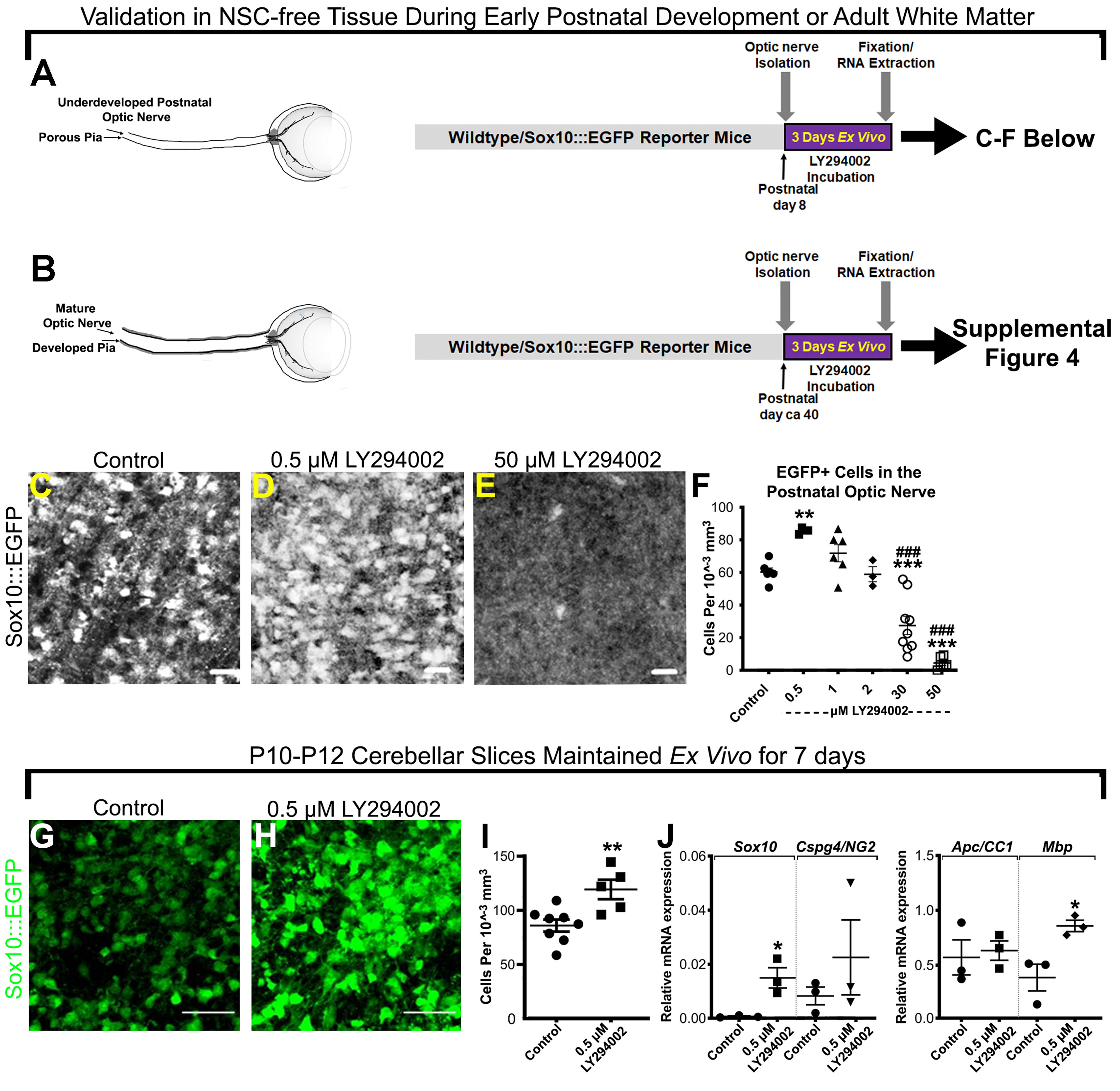

Supplement: Supplementary file 4 — Supplemental Fig. 3: LY294002 regulates oligodendroglial cells in the postnatal optic nerve and cerebellar slices ex vivo. (A-B) Schematic overview of optic nerve growth from postnatal periods to adulthood and timelines used for assessing the impact of LY294002 on OL differentiation. (C-F) Representative confocal images of optic nerves from postnatal Sox10-EGFP mice maintained for 3DIV, in control medium (DMSO), and medium containing increasing concentrations of LY29. Confocal micrographs of the whole-mounted optic nerve illustrates concentration- dependent effects of LY29 in the adult optic nerve. (F) Histogram of mean ( ± SEM) cell counts per constant FOV (n = 3 for each group); * * p < 0.01, * ** p < 0.001 (comparing controls to treatment groups), ####p < 0.0001, (comparing LY-29 0.5 µM to higher LY29 concentrations), One-way ANOVA followed by Dunnett’s multiple comparisons test. (G, H) Representative confocal images (captured via a x20 objective) of cerebellar slices from postnatal Sox10-EGFP mice maintained for 7DIV in 0.5 µM LY29-containing medium; scale bars = 100 µm. (I) Histogram of mean ( ± SEM) cell counts per constant FOV (n = 5 for each group). * * p < 0.01, two-tailed unpaired t-test. (J) Ex vivo cerebellar slices were maintained in 0.5 µm LY29 or control vehicle (saline/DMSO) conditions for 3DIV and tissue harvested for processing for qPCR for oligodendroglial transcripts. Data are normalised to the housekeeping gene GAPDH (ΔCt) and expressed as mean 2-ΔCt ± SEM (n = 3 for each treatment group). * p < 0.05, two-tailed unpaired t-test. [file mmc4.jpg]

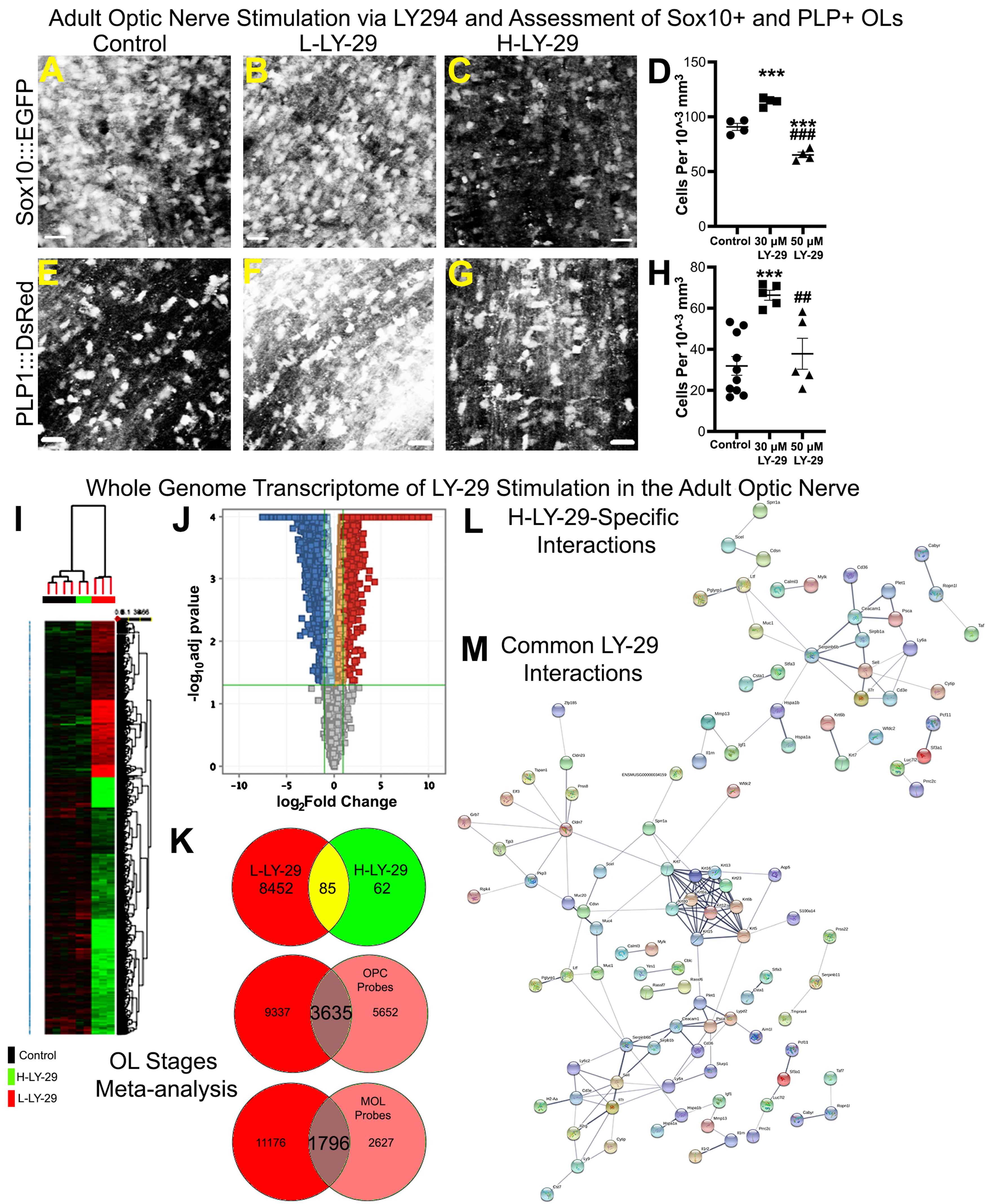

Supplement: Supplementary file 5 — Supplemental Fig. 4: Concentration-dependent effects of LY29 on adult optic nerve oligodendroglia and whole genome profiling for revealing genes regulated by LY29. (A-C) Representative confocal images (captured via a x20 objective) from Sox10-EGFP (top panels) and PLP-DsRed1 (lower panels) optic nerves maintained in culture for 3DIV in control (DMSO) (A), 30 µM LY29 (B), or 50 µM LY29 (C); scale bars = 20 µm. (D) Histogram of mean ( ± SEM) cell counts per constant FOV (n = 6 for each group), * ** p < 0.001, (comparing vehicle control to all treatments), ##p < 0.01, ####p < 0.0001 (comparing 30 µM LY29 to 50 µM), One-way ANOVA followed by Tukey’s post-hoc test. (H, I) Heatmap of transcriptional changes occurring in the adult optic nerves incubated in control medium and in 30 or 50 µM LY29. Red, green and black represent probes upregulated, downregulated or showing no change, respectively, and the most significant probes shown on a volcano plot in I. (J) Venn diagram’s of probes specific to or common between the concentrations of LY29 (top Venn’s), meta-analysis of OPC-enriched (middle Venn’s) or MYOL-enriched (bottom Venn’s) profiles using publically available OPC- and MYOL-derived datasets (see Materials and Methods). (K, L) Protein interactome networks generated using the STRING database of genes common to both LY29 concentrations or those specific to H-LY29. [file mmc5.jpg]
